# Context-Dependent Preferences in Starlings: Linking Ecology, Foraging and Choice

**DOI:** 10.1371/journal.pone.0064934

**Published:** 2013-05-21

**Authors:** Marco Vasconcelos, Tiago Monteiro, Alex Kacelnik

**Affiliations:** 1 School of Psychology, University of Minho, Braga, Portugal; 2 Department of Zoology, University of Oxford, Oxford, United Kingdom; University of Sussex, United Kingdom

## Abstract

Foraging animals typically encounter opportunities that they either pursue or skip, but occasionally meet several alternatives simultaneously. Behavioural ecologists predict preferences using absolute properties of each option, while decision theorists focus on relative evaluations at the time of choice. We use European starlings (*Sturnus vulgaris*) to integrate ecological reasoning with decision models, linking and testing hypotheses for value acquisition and choice mechanism. We hypothesise that options' values depend jointly on absolute attributes, learning context, and subject's state. In simultaneous choices, preference could result either from comparing subjective values using deliberation time, or from processing each alternative independently, without relative comparisons. The combination of the value acquisition hypothesis and independent processing at choice time has been called the Sequential Choice Model. We test this model with options equated in absolute properties to exclude the possibility of preference being built at the time of choice. Starlings learned to obtain food by responding to four stimuli in two contexts. In context [AB], they encountered options A_5_ or B_10_ in random alternation; in context [CD], they met C_10_ or D_20_. Delay to food is denoted, in seconds, by the suffixes. Observed latency to respond (L_i_) to each option alone (our measure of value) ranked thus: L_A_≈L_C_<L_B_<<L_D_, consistently with value being sensitive to both delay and learning context. We then introduced simultaneous presentations of A_5_ vs. C_10_ and B_10_ vs. C_10_, using latencies in no-choice tests to predict sign and strength of preference in pairings. Starlings preferred A_5_ over C_10_ and C_10_ over B_10_. There was no detectable evaluation time, and preference magnitude was predictable from latency differentials. This implies that value reflects learning rather than choice context, that preferences are not constructed by relative judgements at the time of choice, and that mechanisms adapted for sequential decisions are effective to predict choice behaviour.

## Introduction

We present a theoretical and experimental investigation of animal choice that links foraging ecology, state- and context-dependent learning and hypothetical cognitive mechanisms for choice. Foraging animals search, detect, pursue, catch, and consume prey of diverse kinds, each time experiencing the consequences of the whole cycle of events. Throughout these sequences they store information about prey physical attributes (e.g., search time, size, taste, handling time) and about experienced consequences of their decision to pursue such prey (e.g., hedonic impact, improvements in energetic state or other psychological correlates of survival and reproduction).

From a learning point of view, experienced consequences constitute biologically programmed signals that retrospectively give value to (reinforce) earlier links of each typical sequence (e.g., [1], [2]) and hence affect future foraging decisions. In terms of learning theory, stimuli identifying prey types are conditional stimuli (CS) and the consequences of pursuing each prey type are unconditional stimuli (US). If, in infrequent occasions, the CSs corresponding to different outcomes occur simultaneously, for instance if two prey types are simultaneously in the field of view, the subject faces a choice. In such cases, preference between the stimuli that identify prey types will depend on the history of reinforcement, namely the returns with which each set of stimuli has been correlated in the past (e.g., [3]–[5]). This can produce apparently irrational preferences, because absolute properties of prey types (i.e. context and state independent properties) are not the only determinants of their subjective value. The point we are making for learning can underlie observed irrationalities such as the Concorde (in evolutionary biology) or Sunk Cost (in economics) fallacies [6]–[8] and ‘within-trial contrast’ in experimental psychology [9], [10]. To elaborate this point, consider how an unwarranted preference can be caused by learning history: consuming two prey of equal body mass causes the same energy gain and should be treated indifferently, but if typically one of them has higher searching costs, the costly one would have been experienced under greater need and would elicit a memory for higher hedonic impact [11]–[18], leading to it being preferred. The rationale can be extended to the effect of context. If two prey of equal body mass are typically met in periods where they rank differently respect to the background of alternative prey types, then they can be mnemonically associated with positive (elation) or negative (frustration) contrasts, independently of energetic state. Such contrast effects are well known behaviourally and neurobiologically (e.g., [19]–[21]).

Optimal choice in simultaneous encounters should be free from such effects, but simultaneous encounters may be too infrequent to select for learning processes immune to these fallacious effects. The structure of most species' ecology means that state- and context-dependent reinforcement, evolved as adaptations to serial cycles, are likely to dominate learning-dependent valuation (e.g., [22]–[24]). Thus, we conclude that the subjective valuation of each option may be affected by factors other than their intrinsic objective value because of learning processes but these valuations must interact to control choices [25]–[29], and this we discuss next.

Learning, choice and decision making attract multidisciplinary interest, and it is not surprising that theories follow differing rationales and make different predictions. For instance, a comprehensive recent review ([30]; see also [31], [32]) includes process-specific predictions in the definition of what a decision is, starting thus: ‘A decision is a deliberative process that results in the commitment to a categorical proposition. An apt analogy is a judge or jury that *must take time to weigh evidence* for alternative interpretations and/or possible ramifications before settling on a verdict.’ (p. 536, our italics).

The view that decisions take time and deliberation time reflects a tradeoff between speed of action and accuracy of decision is a logical expectation, but it is still subject to empirical research. We have argued that because sequential encounters are frequent and simultaneous choices rare, the latter are handled by mechanisms selected for their performance in the former, and this may mean an absence of deliberation time. The mechanism we propose is that in the (rare) simultaneous encounters, each option elicits its own typical latency to act reflecting its encoded subjective value, and a “choice” results from responding towards the option eliciting the shorter latency [28].

**Figure 1:**
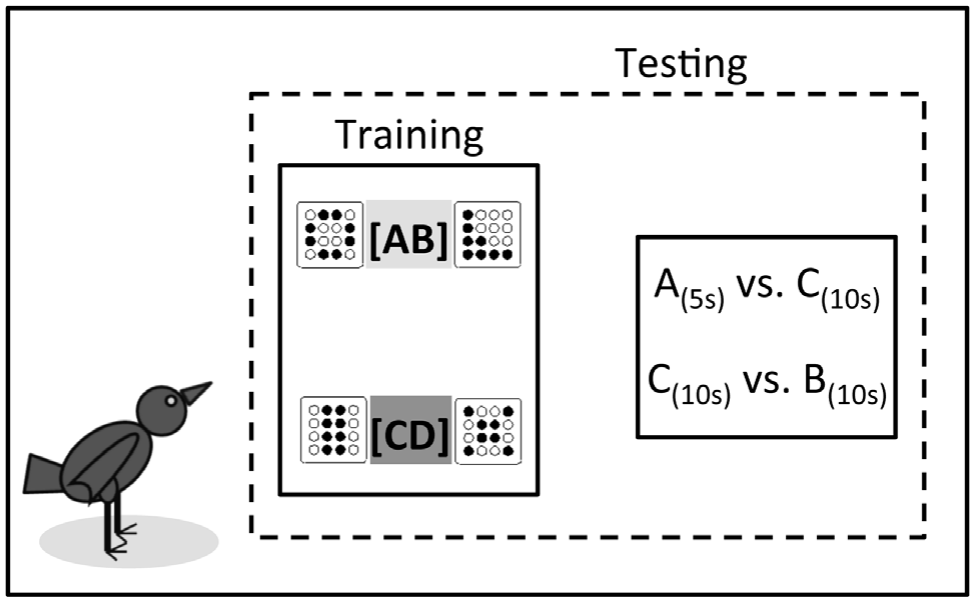
Experimental design schematics. Animals were trained in two alternating contexts. In context [AB], they encountered options A_5s_ or B_10s_ in random alternation; in context [CD], they met C_10s_ or D_20s_ (*Training*). The animals were then tested by introducing, infrequent simultaneous choice trials between options that belonged to different contexts, namely between A_5s_ and C_10s_ and between B_10s_ and C_10s_ (*Testing*). Delay to food in each option is denoted, in seconds, by the suffixes; symbols exemplify the type of stimuli presented.

Our overall view is that subjective valuation reflects state- and context-dependent changes in wellbeing caused by each option in no-choice encounters, and that when options are met simultaneously preferences reflect a horse-racing competition for expression of each option treated independently. To emulate a stylized version of natural foraging, in our experiments the subjects learn by facing different alternatives sequentially, and in infrequent occasions face simultaneous choices between types. In particular, we trained European starlings (*Sturnus vulgaris*) in two contexts, [AB] and [CD], thus generating context-dependent differential utilities across foraging options [33], [34]. In context [AB], they sequentially encountered options A_5_ or B_10_ in random alternation; in context [CD], they met C_10_ or D_20_ in a similar fashion. Suffixes indicate delays to food, in seconds. The animals then faced infrequent simultaneous choice trials between options that belonged to different contexts, namely between A_5_ and C_10_ and between B_10_ and C_10_ (Figure 1).

We make the following specific predictions: (i) in sequential encounters stimuli whose outcome causes more favourable state changes relative to their context will cause shorter latencies to respond. Notice that options equal in absolute properties but experienced in different contexts can then have different value; (ii) preferences in simultaneous encounters should be predictable by assuming cross-censorship between the latencies observed in sequential encounters, and (iii) decisions should not take extra time; responding to an option in a simultaneous choice should not take longer than when the same option is encountered alone.

## Materials and Methods

### Ethics statement

The study was approved and carried out in strict accordance with the recommendations of the Ethical Review Committee of the Department of Zoology of the University of Oxford. The starlings that participated in this experiment were obtained and kept under Natural England license No. 20083718 and were reintroduced into a communal aviary after the experiment, before being released back into the wild. All efforts were made to minimize suffering.

### Subjects

Eight wild-caught adult European starlings (*Sturnus vulgaris*) participated in the experiment. All had laboratory experience unrelated to the contingencies they encountered here. The birds were kept outdoors before starting the experiment, when they were transferred to individual indoor cages (160 cm×45 cm×40 cm) that served as housing and testing chambers. Indoor temperatures ranged from 15 to 18°C and lights followed a 12∶12 dark schedule with light from 0700 to 1900. Starlings were rewarded with 20 mg BioServ® precision pellets throughout the experimental sessions and provided with three hours of free access to turkey crumbs (14∶30 to 17∶30) and 10 mealworms daily. This regime allows the starlings' body weights to remain stable at approximately 90% of their free-feeding weight [35].

### Apparatus

Each cage had an operant panel attached to the centre of the back wall. The panel had three sections: a middle subpanel, facing the cage (10 cm wide), and two side subpanels (each 10 cm wide) attached to the cage at a 110 degree angle from the centre subpanel. Each panel had a response key in the centre (11 cm from the bottom) that could be illuminated with a variety of hues and black and white symbols. The middle subpanel also had a food hopper (2 cm from the bottom) that was connected to a pellet dispenser (Campden Instruments®). Trials were controlled by custom software running on the Microsoft® Windows® operating system and attached to an Animal Behavior Environment Test System (Campden Instruments®) via WhiskerServer [36].

### Procedure

#### Training

After brief preliminary training (detailed in Text S1) the training phase began. All subjects were exposed to four stimuli (options), implemented as coloured keys that, once pecked, led to a food reward being delivered after a pre-established waiting time, or delay. Two of the options were encountered in one context and the other two in a different context. Contexts are defined exclusively by the pair of options that could potentially be encountered. In context [AB] there were random sequential encounters with options A_5_ or B_10_ and in context [CD] with options C_10_ or D_20_.

Starlings received one training session per day for 12 days. Each session was divided into four 44-trial sub-sessions, each sub-session involving one of the two contexts, yielding up to 176 trials per day/bird (thus a maximum of 7.04 g of food could be received during testing by each subject). The order of presentation of contexts alternated across subjects and days. Half of the subjects started the first session with context [AB] and the other half with context [CD]. On each particular session, the subjects that began with context [AB] ended with context [CD], and on the next day, they began with context [CD] and ended with context [AB]. Contexts were separated by an interval of either 45 (first and third transition) or 60 minutes (second transition). Sessions ended after 176 trials or 6.5 hours from the session start, whichever came first.

Within each context, birds experienced 40 single-option trials (20 per option) and 4 peak trials (2 per option), all separated by a 30-s inter-trial-interval (ITI). Single-option trials emulated sequential encounters in natural settings and provided the birds information about the properties of each option. These trials began with the green centre key flashing (700 ms ON, 300 ms OFF). A single peck to this key switched its light off and caused one of the side keys to start flashing one of four possible symbols at the same rate used for the centre key. A peck to this key turned it steadily on and initiated the delay associated with the displayed symbol (5, 10 or 20 s). The time between pecking the green centre key and pecking the flashing side key is hereafter referred to as the latency to respond. The first peck after the delay elapsed turned the side key symbol off and produced two food pellets followed by the ITI. Peak trials were identical to single-option trials except that once the flashing side key was pecked, the symbol remained continuously on for 60 s, and then extinguished without reinforcement. These trials were used to determine the animals' knowledge of the delay to reward associated with each particular option [37], [38]. The order and sides in which the options were presented were pseudo-randomised with the constraint that no two-peak trials could occur consecutively. Symbol/delay parings were partially counterbalanced across birds. To equate the rate of reward across contexts and thus minimize possible differences in satiation states, we adjusted the usual 30 s ITI every fifth single-option trial. We proceeded as follows: we summed the delays to food from the preceding five single-option trials, subtracted this value from its theoretical maximum of 100 s (5 delays of 20 s) and then added this value to the 30 s ITI.

#### Testing

This phase was structurally similar to the training phase except that choice trials were added. All such trials were cross-context, involving choices either between A_5_ and C_10_ or between B_10_ and C_10_. Half of the subjects were first tested with choices between A_5_ and C_10_ and the remaining half with choices between B_10_ and C_10_. When preference reached stability, choice types were swapped: subjects that previously chose between A_5_ and C_10_ faced choices between B_10_ and C_10_, and vice-versa. Both tests continued until the stability criteria were met: a standard deviation less than 10 for choice percentages and no visible trend in choice percentages, both during the last three sessions.

Sixteen simultaneous choice trials were presented daily. These were presented in pairs and separated by the ITI. Each pair of choice trials was presented at the beginning and the end of each context substituting the first and last two single-option trials that would normally occur during training. Choice trials were similar to single-option trials with the exception that pecking the flashing centre key caused two different symbols, one on each side key, to begin flashing simultaneously. A single peck to one of these keys turned the selected symbol continuously on, triggered the appropriate delay, and extinguished the unselected option. Thus, a bird committed itself to one of the options with its first peck to a flashing symbol. Again, the time between pecking the centre key and pecking one of the flashing side keys will be considered the as latency to choose. Once an option was selected, the trial proceeded as a regular single-option trial.

#### SCM simulations

Only latencies from single-option trials collected at stability were used to run the simulations. To compute predictions for preferences of A_5_ vs. C_10_ and of B_10_ vs. C_10_, we used latencies from single-option trials in the same test sessions in which choice proportions were measured. A separate Monte Carlo simulation using a nonparametric estimate of the cumulative distribution functions of the data yielded extremely similar predictions and thus is not reported.

Each simulation involved 10,000 experiments of 96 trials each [48 A_5_ vs. C_10_ and 48 B_10_ vs. C_10_ trials]. For any given trial, the simulation drew one latency for each option [A_5_ and C_10_ or B_10_ and C_10_] from the population of observed latencies during single-option trials and predicted the option to be chosen in the simultaneous presentation as the one yielding the shortest latency.

#### Data analysis

Prior to analysis, all proportion and latency data were successfully normalized using an arcsine square root and a natural log transformation, respectively [39], [40]. A Type-1 error rate of 0.05 was adopted for all reported statistical comparisons. Preliminary analyses (see Text S2 for details) revealed that the context in which test trials occurred had no significant effect on preference, thus we pooled data across testing contexts for all further analyses. One bird was dropped early on from the experiment due to showing a strong side preference.

## Results

### Preferences

**Figure 2:**
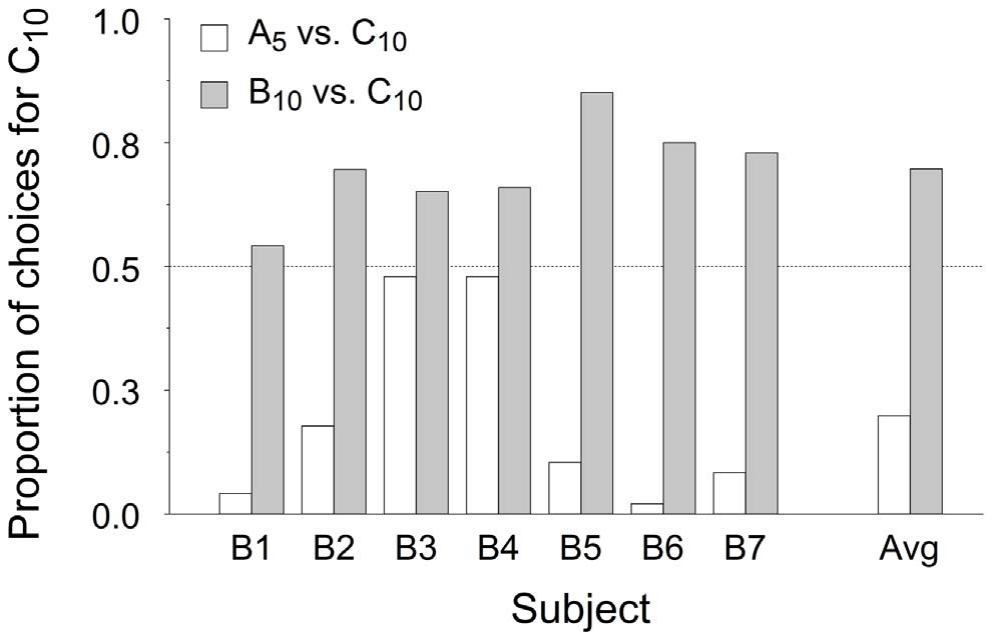
Proportion of choices for option C_10_ in both preference tests. White and grey bars show preference for C_10_ in the A_5_ vs. C_10_ and B_10_ vs. C_10_ tests, respectively. The rightmost columns show average data. The horizontal dashed line indicates chance level

When given a choice between two options with the same within-context ranking but signalling different delays to reward [A_5_ vs. C_10_], starlings chose C_10_, the option with lower immediacy, 19.8% of the time (range: 2.1–47.9%). Conversely, when immediacy was the same but within-context ranking differed [B_10_ vs. C_10_], starlings preferred the option with higher ranking, C_10_, 69.7% of the time (range: 54.2–85.1%). One-sample *t* tests of magnitude of preference against indifference revealed that both these findings deviated significantly from chance [*t*(6) = −3.828, *p* = .009 and *t*(6) = 5.086, *p* = .002 for the A_5_ vs. C_10_ and B_10_ vs. C_10_ tests, respectively]. Figure 2 shows both average and individual choice proportions.

### Perception and encoding of delay to reward

**Figure 3:**
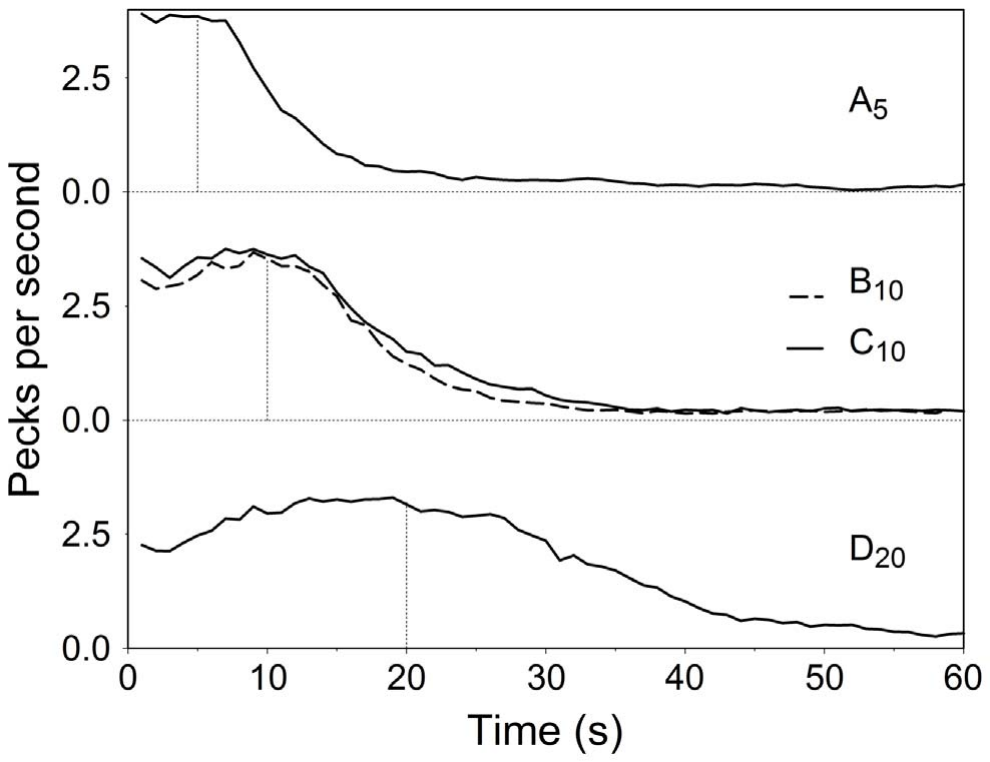
Mean pecks per second in peak trials for options A_5_, B_10_, C_10_ and D_20_ The vertical dashed lines indicate the time at which reward was due in rewarded trials.

A candidate explanation for the preferences observed in the B_10_ vs. C_10_ tests lies in possible distortions of memory for waiting times. If the representation of the temporal properties of each option (i.e., their immediacy) is affected by its context, then the starlings may in fact be choosing between options that, in spite of being objectively equivalent, are remembered as signalling subjectively different delays to reward. We used two techniques to test this hypothesis: one based on pecking patterns and the other based on peak time (i.e., the time with maximum response rate). Figure 3 shows the pecking pattern in peak trials, averaged across subjects. For all options, the average maximum response rates were near the programmed reward time while peak rates decreased and spreads increased with longer delays. A repeated-measures analysis-of-variance (ANOVA) with option and time as fixed factors, subjects as random factor and pecking rate as dependent variable confirmed a significant effect of option [*F*(3,18) = 42.629, *p*<.001], time [*F*(59,354) = 121.546, *p*<.001] and their interaction [*F*(177,1062) = 18.919, *p*<.001]. Of particular interest are the pecking patterns for options B_10_ and C_10_ as they provide a test for the hypothesis that contextual effects on preference were mediated by distortions of subjective temporal estimates. Post-hoc pairwise comparisons revealed that pecking rates did not differ systematically between options B_10_ and C_10_ (*p* = .289), but did differ for all other comparisons (largest *p* = .018), thus suggesting that the effect of within-context ranking was not mediated by distortions in interval timing. The analyses based on the location of peaks returned similar findings (see Text S3 for details).

### Sequential-Choice Model

#### Simulations

**Figure 4:**
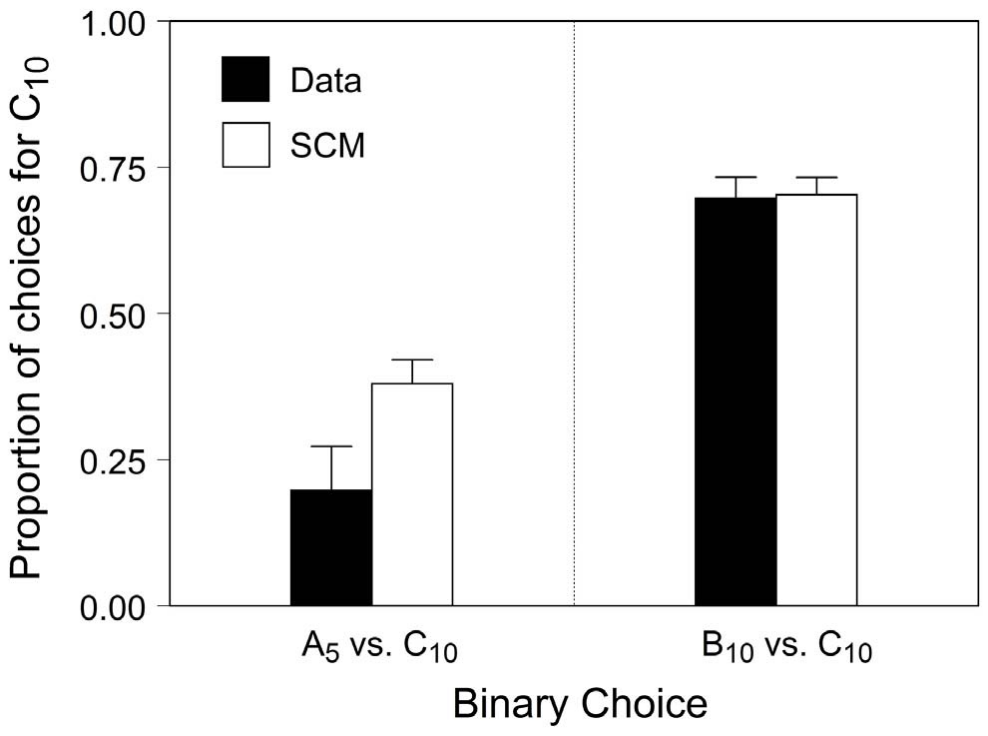
Observed and predicted proportion of choices for option C_10_ in both preference tests. Black bars represent the observed average preference for C_10_ in the A_5_ vs. C_10_ (left) and B_10_ vs. C_10_ (right) preference tests. White bars represent the preference for C_10_ predicted by the Monte Carlo simulations in the same tests. Error bars represent one SE.

**Figure 5:**
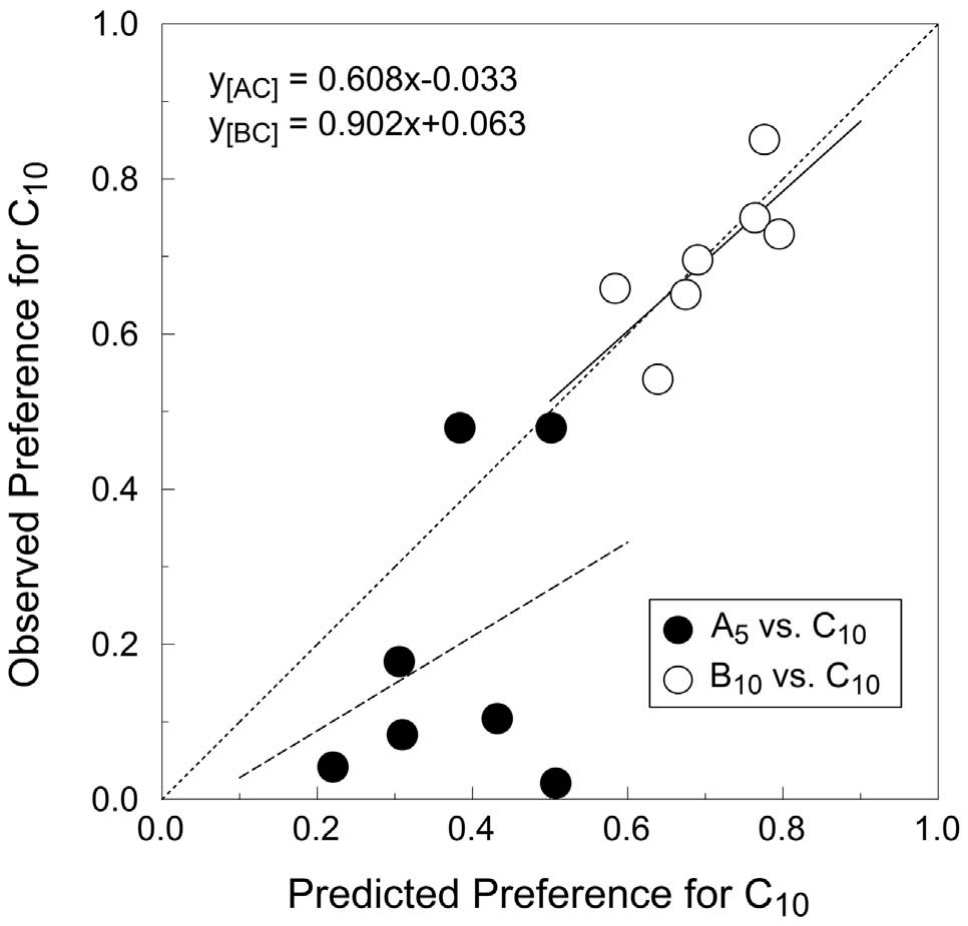
Overall fit of the SCM. Scatter plot of the preference for C_10_ predicted by the SCM versus the observed preference for C_10_. Each dot represents one subject, with black and white dots referring to the A_5_ vs. C_10_ and B_10_ vs. C_10_ tests. Both lines are linear regressions.

According to the SCM, preferences in simultaneous choice trials should be predictable from the latencies observed in sequential encounters. Predicting choice preferences from performance in single-option (no-choice) trials is an exclusive feature of the SCM, and for this reason it is of paramount importance for this study. The simulations predicted average preferences for C_10_ of 37.9% and 70.3% in A_5_ vs. C_10_ and B_10_ vs. C_10_ tests, respectively. Figure 4 shows the observed preferences (black bars) and the predictions of the simulations (white bars). Qualitatively, the simulation predicted both kinds of choices accurately. However, while the SCM predicted the results of the B_10_ vs. C_10_ tests with high accuracy, it did not do so for the A_5_ vs. C_10_ tests, where observed preferences were more extreme than those predicted. Figure 5 shows the observed versus predicted preferences for C_10_ for all subjects in both tests as well as an unconstrained regression line for each test. The best case model performance occurs when every data point falls in the main diagonal (where predictions of the model and empirical observations match). For the B_10_ vs. C_10_ tests, the regression line falls close to the theoretical optimum whereas for the A_5_ vs. C_10_ tests the regression deviates considerably. One way to read the results of the A_5_ vs. C_10_ tests in Figure 5 is that, based on the latencies of sequential trials, the simulations never predict preferences for A_5_ stronger than 80%, but the data do show such cases. We will refer to a potential explanation for this dissociation in the ability of the SCM to quantitatively predict choice across tests in the discussion.

#### Latencies in sequential encounters

**Figure 6:**
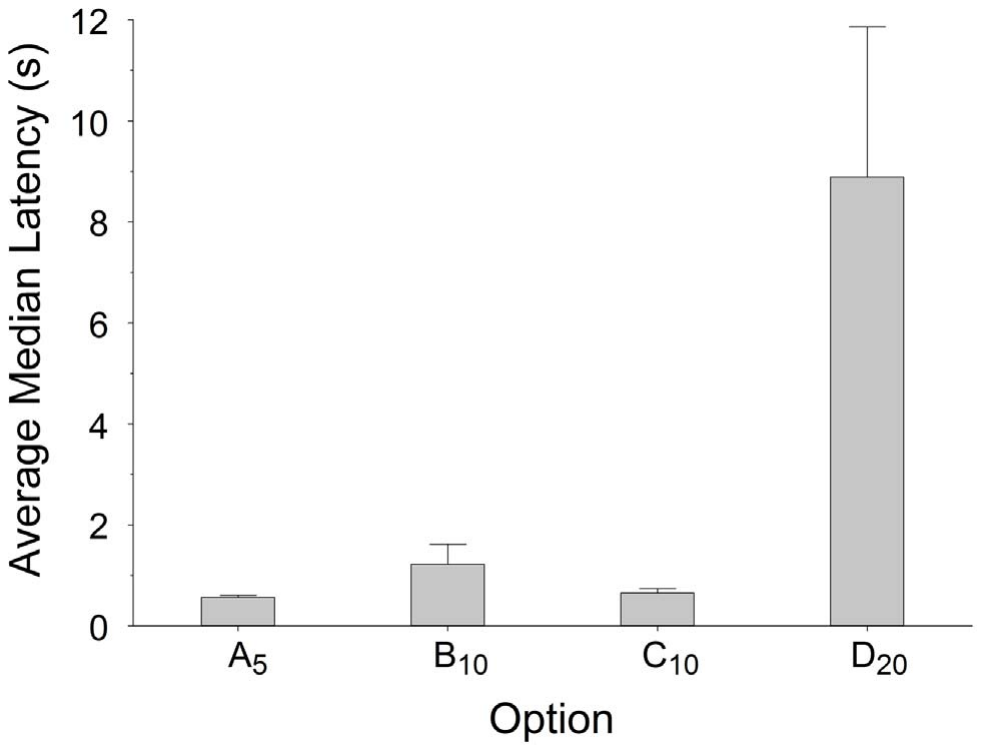
Latencies to accept each option in single-option trials. Median latencies to accept options A_5_, B_10_, C_10_ and D_20_ in single-option trials, averaged across subjects (+SE).

The SCM argues that latencies to respond to opportunities encountered sequentially reflect both the attributes of each option and those of the background opportunities, and that such latencies should be good predictors of the relative preferences of options when encountered simultaneously. Here we test the first part of these arguments. Figure 6 shows the average median latencies for each option during single-option trials. A repeated-measures ANOVA conducted on these data confirmed a significant effect of immediacy [Greenhouse-Geisser adjusted *F*(1.236, 7.417) = 20.042, *p* = .002]. Post-hoc pairwise comparisons showed that latencies differed significantly between options (largest *p* = .019) except for the comparison between options A_5_ and C_10_ (p = .081). Further, these latencies reflected both the immediacy and ranking of each option in its own context: greater immediacy and better ranking both shortened latency to accept an option. In particular, the within-context ranking effect is demonstrated because latencies to accept option C_10_ were significantly shorter than latencies to accept option B_10_ despite both options signalling the same delay to reward. In contrast, despite A_5_ and C_10_ signalling different delays in this case the difference between median latencies was short of conventional significance. These findings show that ranking did participate in the valuation of the options. The absence of a conventionally significant difference between A_5_ and C_10_, does not imply that absolute attributes do not have any effect, because these latencies were sufficiently short to approach psychophysical reaction times (cf. [41]) rather than strategically variable delays to accept individual options. If a subject's response is close to its reaction time, “floor effects” are likely to mask any possible difference in subjective value.

Since there is a danger that an analysis based on medians may miss a potential effect of differences in the tails of the distributions, we also performed an analysis based on the whole frequency distributions of latencies (see Text S4 and Figure S1). This analysis showed the same trends. In sum, the latencies collected during single-option trials confirm the SCM's proposal that such latencies should be sensitive both to the option's objective properties (i.e. its immediacy) and to the properties of the background alternatives available in the environment (i.e. its ranking).

#### Latencies in simultaneous choices

The comparison between latencies in single-option and choice trials pitches models based on deliberative comparison at the time of choice and on independent processing of options against each other: executing a cognitive comparison should make simultaneous-choice latencies longer than single-option latencies, while cross-censorship in the horse-racing SCM model should lead to choices taking less time than single option responses. The magnitude of the expected shortening depends on the similarity between the distributions of latencies collected during single-option trials, and shortening should be greater for the option that is less preferred (i.e., more censored) in choice trials.

**Figure 7:**
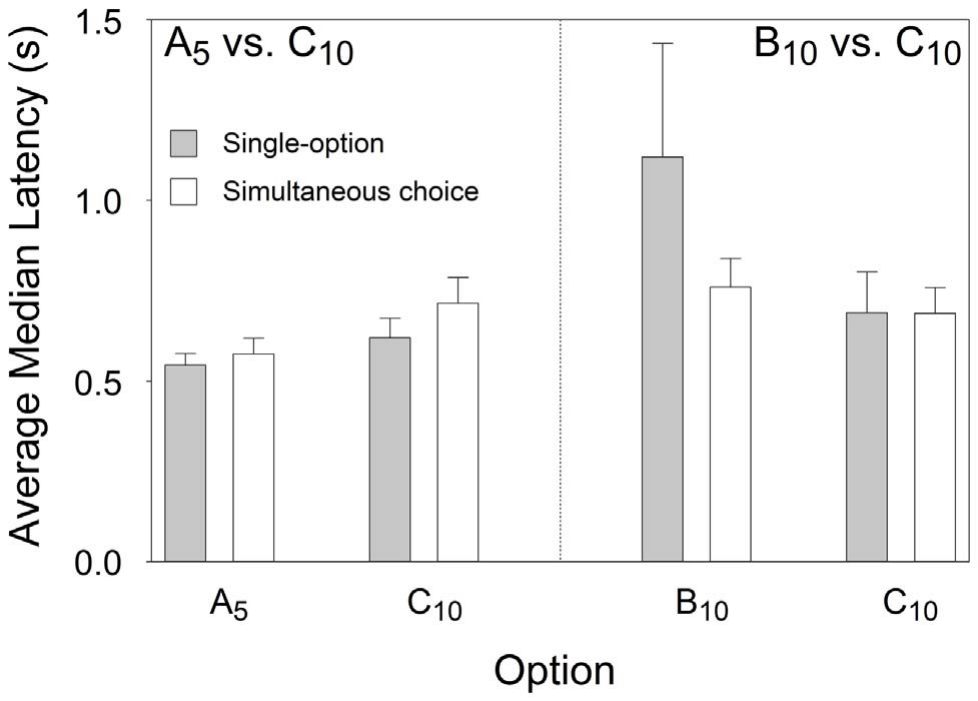
Latencies to accept each option in single-option and simultaneous choice trials. Latencies are separated according to collection time: either at stability in the A_5_ vs. C_10_ preference tests or at stability in the B_10_ vs. C_10_ preference tests. Grey and white bars present median latencies averaged across subjects (+SE) from single-option and simultaneous choice trials, respectively.

Figure 7 shows the average median latencies for each option when chosen in simultaneous choice trials and when encountered alone in single-option trials. Paired-samples *t*-tests failed to reveal significant difference in latencies depending on whether an option was encountered by itself or chosen in a simultaneous choice situation [all *t*s(6) ≥−1.577 and ≤1.326, smallest *p* = .166].

This central tendency analysis was complemented by analyses of the complete frequency distributions of latencies (see Figure S2 for details). We summarized each individual latency distribution by means of a cumulative frequency score of latencies both in forced and simultaneous choice trials using the approach described in Text S4. We also calculated such scores for choice latencies in the SCM Monte Carlo simulations. The latter predicted a significant shortening only for option B_10_ [*t*(6) = 2.571, *p* = .042], but the observed shortening for this option, while having the expected trend, did not reach conventional statistical significance in a two-tail test [*t*(6) = 2.155, *p* = .075], even though it was the strongest shortening we observed (see panel B, Figure S2). We are aware that given the strength of the *a priori* directional prediction towards a shortening a one-tail test might have been considered more appropriate, but we chose to take the same conservative approach that we used throughout and treat this result as suggestive but not robustly demonstrated. All other predicted and observed shortenings were non-significant. No lengthening of latencies (which would support a deliberative comparison model) was either predicted by the Monte Carlo simulations or observed for any option.

## Discussion

Our main objectives were to integrate ecological reasoning, knowledge about learning, and concepts of decision processes to advance towards a comprehensive model of foraging choices. We tested predictions about learning effects (basically that subjective value as acquired in no-choice encounters depends on state and context-dependent effects of each option's outcome) and whether the translation of these subjective values into preferences in simultaneous encounters involved a relative comparison at the time of choice or resulted from the expression of the same mechanisms used in no-choice encounters. We found support for our learning hypotheses because when facing simultaneous choices between options differing in either delay to reward or in within-context ranking, starlings' choices were better explained by remembered context-dependent utility than by remembered objective properties. Analysis of temporal responding showed that preferences were not driven by distorted memory for temporal properties of the options.

Consistently with the assumptions of the SCM, latencies to accept options presented singly were affected both by the options' objective properties and their within-context ranking: the higher the immediacy and the better the ranking the shorter the corresponding latencies. This supports the view that latencies expressed in sequential encounters reflect the operation of a valuation process and justifies their use in predicting simultaneous choice [25]–[29].

One of the unique predictions of the SCM is that we ought to be able to predict preferences in simultaneous choices from latencies observed during sequential encounters. The present experiment tested this prediction in a situation where valuation was context-dependent and thus significantly influenced by background alternatives. The model was qualitatively accurate in both choice tests and remarkably accurate quantitatively in one of them. Specifically, the model predicted preferences in simultaneous choices were almost identical to those observed when the options have the same immediacy and different rankings [B_10_ vs. C_10_] but were directionally correct but less extreme than observed when options had the same ranking and different immediacies [A_5_ vs. C_10_]. This pattern of findings relates to our latency findings: both the analysis based on median latencies and that based on cumulative frequency distributions indicated that the latencies recorded for A_5_ in single-option trials were somewhat shorter (but not reliably so) than those found for C_10_. Thus, the results from the Monte Carlo simulations (that used latencies from the same distributions) did not reach the strength of preference for A_5_ that was observed, despite predicting preference in the correct direction. Conversely, we did find significant differences between the distribution of latencies for B_10_ and C_10,_ which in turn yielded predictions that virtually matched the observed preferences.

What could have caused this differential success? The SCM's assumption is that differences in subjective value of an option relative to its background alternatives are detectable in the readiness to respond to it, expressed as a distribution of latencies to accept single options. The model does not claim that latencies cause choices but instead that latencies and choices are expressions of a common process acting both in sequential and simultaneous choice [28]. Tests of the SCM use latencies as ‘windows’ to the processes involved in the subjective valuation of each option. When there are clear differences in latency distributions such as observed between options B_10_ and C_10_ (cf. Figure S1) the model predicts preference very accurately. When latency distributions mostly overlap, such as for options A_5_ and C_10_ (cf. Figure S1), the model is unable to generate extreme preferences such as those obtained in the A_5_ vs. C_10_ preference tests.

Clearly, given that in the case of A_5_ vs. C_10_ preferences were very strong but latencies did not differ much, comparable latency distributions are not diagnostic of equivalent subjective value. We believe that this may have been an expression of a floor effect. The average median latencies for options A_5_ and C_10_ during sequential encounters were .565 and .654 sec, respectively, even though they had a 2∶1 ratio in terms of immediacy. These extremely short response latencies together with the starlings' sensitivity to their immediacy [viz., they strongly preferred A_5_ in the A_5_ vs. C_10_ preference tests] suggest that latencies to accept these options were levelled by reaction times. The high subjective value of both options led to latencies so short as to reach the limits of reaction time in the present apparatus. The fact that the birds can generate strong choice preferences in the absence of differences in latencies argues for the existence of routes to drive choice other than those embodied in the SCM, but it would appear that these additional routes only kick in when latencies are very short and their distributions superimpose.

We also pointed out that while the conventional deliberative models of choice predict a lengthening of latencies in choices versus no-choice encounters, the SCM predicts the opposite [25], [28]. Our simulations of the SCM choice mechanism indicated that a shortening should be observed when comparing latency to B_10_ in single-option trials and when in the B_10_ vs. C_10_ simultaneous-choice trials. As previously stated, the shortening should be particularly noticeable for the poorer options, as indeed it was. Conversely, options A_5_ and C_10_ elicited short, virtually undistinguishable latencies during single-option trials, making any change in latency between sequential and choice trials very difficult to detect.

The experimental results yielded a shortening, albeit not conventionally significant in a two-tailed test (p = 0.075). In no case was there any indication for an increase in latencies consistent with a deliberation process at the time of choice. Taken together these results favour the SCM cross-censorship mechanism rather than of a deliberative process at the time of choice, particularly because the task both families of models face is asymmetric: latencies have floor but not ceiling and thus lengthening should be easier to observe than shortening.

In short, then, the confluence of ecological and mechanistic hypotheses that we endorse argues that because in natural circumstances animals typically meet feeding opportunities sequentially and seldom simultaneously, their learning and decision processes do not include cognitive mechanisms evolved due to their performance in simultaneous choice, and our observations endorse this view. In sequential encounters animals assign subjective value to prey types according to their state and the contrast with the background, and in simultaneous encounters, when by necessity state and background are unique, the learning past still controls choice. Preference thus is not built at the time of choice, by a cognitive deliberation that evaluates options' differences, but by the shadow of past state-dependent valuation learning.

## Supporting Information

Figure S1 **Average cumulative frequency distribution of latencies for each option during sequential encounters.** The greater the area below each function the shorter the overall latencies to accept that particular option.
(TIF)

Figure S2 **Average cumulative frequency distribution of latencies for each option in single-option and simultaneous choice trials.** Triangles and dots represent data from single-option and simultaneous choice trials. Latencies are separated according to collection time: either at stability in the A_5_ vs. C_10_ preference tests or at stability in the B_10_ vs. C_10_ preference tests. (A) Distributions for A_5_ in the A_5_ vs. C_10_ preference tests. (B) Distributions for B_10_ in the B_10_ vs. C_10_ preference tests. (C) Distributions for C_10_ in the A_5_ vs. C_10_ preference tests. (D) Distributions for C_10_ in the B_10_ vs. C_10_ preference tests.
(TIF)

Text S1 **Preliminary training.**
(PDF)

Text S2 **Preliminary analyses.**
(PDF)

Text S3 **Analyses of peak location.**
(PDF)

Text S4 **Analyses of complete latency distributions.**
(PDF)
