# Supplementary figures and images for: Context-Dependent Preferences in Starlings: Linking Ecology, Foraging and Choice
Source: PLoS One. 2013 May 21;8(5):e64934. doi: 10.1371/journal.pone.0064934 (PMC3660320; doi:10.1371/journal.pone.0064934)

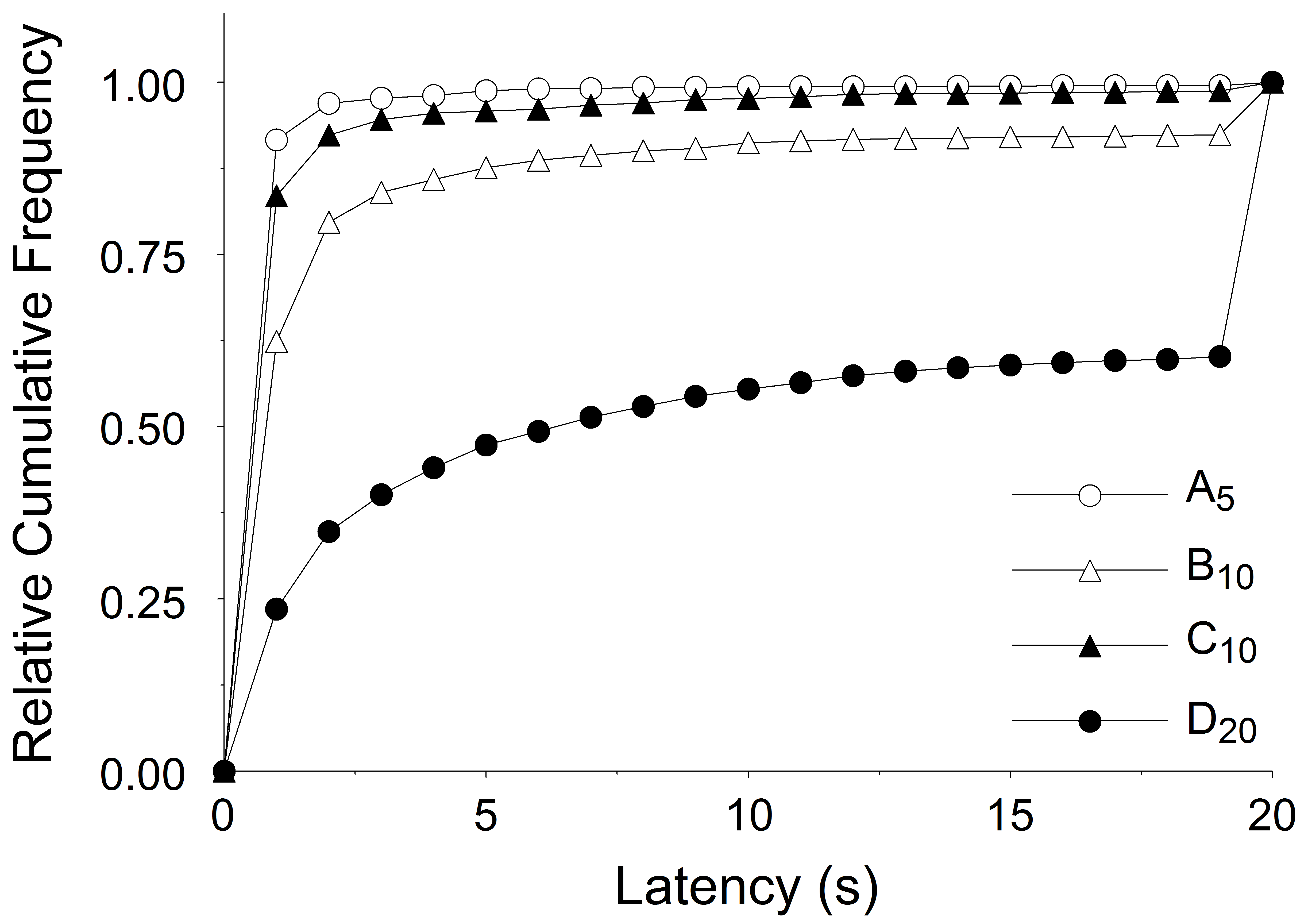

Supplement: Figure S1 — Average cumulative frequency distribution of latencies for each option during sequential encounters. The greater the area below each function the shorter the overall latencies to accept that particular option. (TIF) [file pone.0064934.s001.tif]

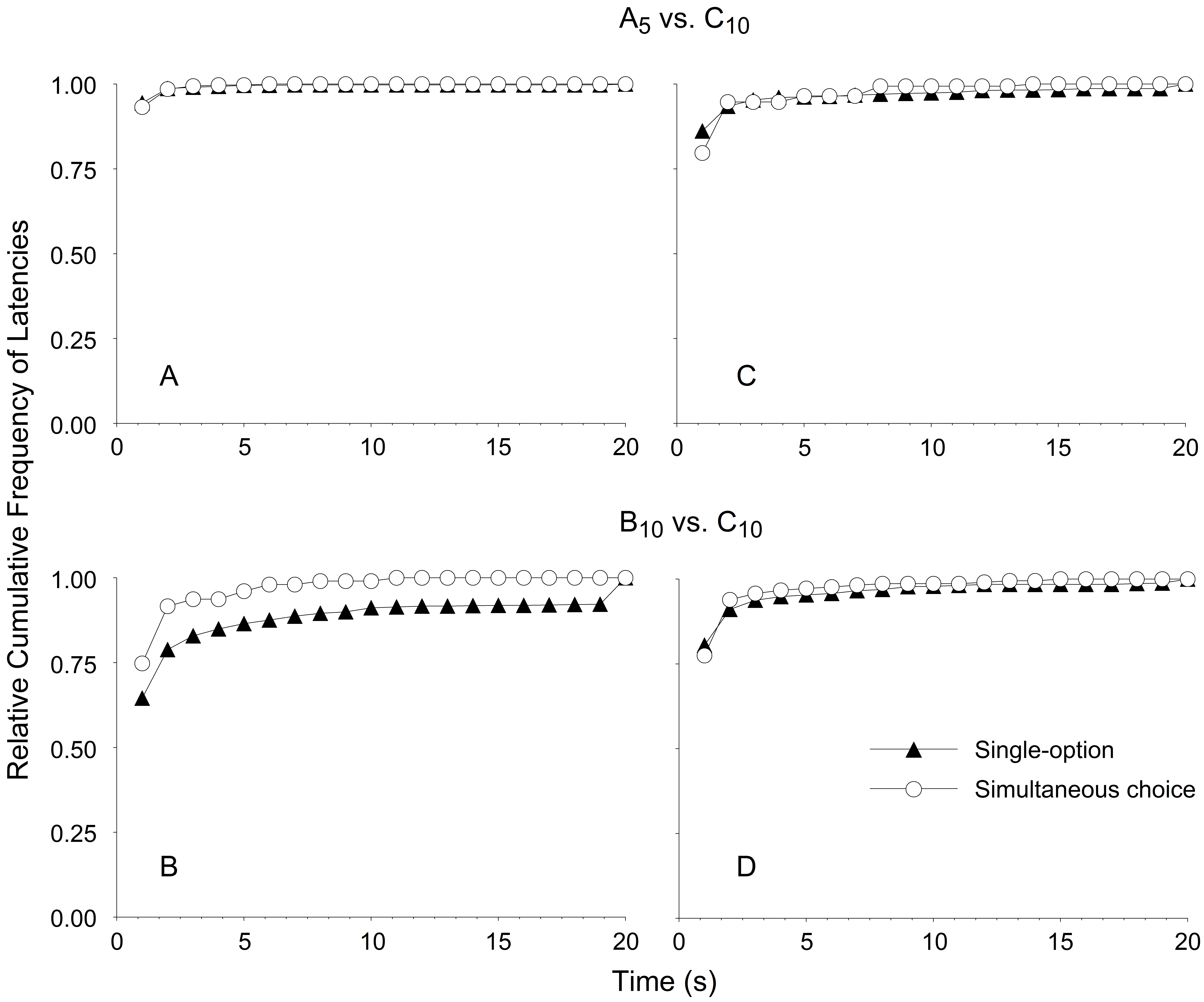

Supplement: Figure S2 — Average cumulative frequency distribution of latencies for each option in single-option and simultaneous choice trials. Triangles and dots represent data from single-option and simultaneous choice trials. Latencies are separated according to collection time: either at stability in the A5 vs. C10 preference tests or at stability in the B10 vs. C10 preference tests. (A) Distributions for A5 in the A5 vs. C10 preference tests. (B) Distributions for B10 in the B10 vs. C10 preference tests. (C) Distributions for C10 in the A5 vs. C10 preference tests. (D) Distributions for C10 in the B10 vs. C10 preference tests. (TIF) [file pone.0064934.s002.tif]
