# Supplementary material for: Context-Dependent Preferences in Starlings: Linking Ecology, Foraging and Choice
Source: PLoS One. 2013 May 21;8(5):e64934. doi: 10.1371/journal.pone.0064934 (PMC3660320; doi:10.1371/journal.pone.0064934)
Supplement: Text S1 — Preliminary training. (PDF) [file pone.0064934.s003.pdf]

Text S1

Preliminary Training. After magazine training, the birds were trained to peck a flashing green centre key (700ms ON, 300ms OFF). A single peck to this key delivered four precision pellets, extinguished the key light, and initiated a 60-s inter-trial-interval (ITI). Failing to peck the key within 30-s from the onset of flashing resulted in the same events as a peck. Training thus combined autoshaping with an operant response. Once birds were reliably pecking the centre key, the lateral keys were introduced. Trials again began with the flashing green centre key. A peck to this key turned its light off and caused one of the side keys to start flashing with a designated black and white symbol (not used in subsequent phases). A peck to the flashing lateral key turned it off and produced two food pellets followed by a 60-s ITI. During this phase, failing to peck the centre key within 30 s from trial onset resulted in a 10-s blackout followed by the ITI. Failing to peck the flashing side key during the same time period was followed by food 50% of the time. This program was maintained 5 hours per day until the birds reliably pecked the centre and side keys on at least 80% of the trials. Next, birds received preliminary, response-initiated fixed interval (FI) training. In this phase two pecks to the side key were required to deliver food. The first peck turned the symbol steadily on and began a delay, the duration of which was progressively increased from 1 to 20 s. The first peck after the delay lapsed extinguished the symbol, delivered two food pellets and initiated a 30-s ITI. Once birds were reliably obtaining food on FI 20 s trials, the experimental sessions began.
