# Supplementary material for: Context-Dependent Preferences in Starlings: Linking Ecology, Foraging and Choice
Source: PLoS One. 2013 May 21;8(5):e64934. doi: 10.1371/journal.pone.0064934 (PMC3660320; doi:10.1371/journal.pone.0064934)
Supplement: Text S2 — Preliminary analyses. (PDF) [file pone.0064934.s004.pdf]

Text S2

Preliminary analyses. Preliminary analyses revealed that neither the context in which simultaneous choices occurred nor the order of testing had a significant effect on preferences either on the A_5 vs. C_{10} [t(6)=.503, p=.633 and t(5)=-.764, p=.479, respectively] or on the B_{10} vs. C_{10} tests [t(6)=-.827, p=.440 and t(5)=-.825, p=.447, respectively]. Similarly, no effect on the number of sessions to reach criterion performance on either test block was observed. On average, the starlings took 6.71 and 7.71 sessions to stabilize preferences on the first and second test blocks, respectively [t(6) = -.635, p=.549] and 6.29 and 8.14 sessions to reach criterion on the A_5 vs. C_{10} and B_{10} vs. C_{10} tests, respectively [t(6) = -1.291, p=.244].
