# Supplementary material for: Context-Dependent Preferences in Starlings: Linking Ecology, Foraging and Choice
Source: PLoS One. 2013 May 21;8(5):e64934. doi: 10.1371/journal.pone.0064934 (PMC3660320; doi:10.1371/journal.pone.0064934)
Supplement: Text S3 — Analyses of peak location. (PDF) [file pone.0064934.s005.pdf]

Text S3

Analyses of peak location. To further explore the time distortion hypothesis we analysed the peak location (i.e., the 1-s time bin of maximum response rate) for options B_{10} and C_{10}. We determined the time bin yielding the maximum response rate for each trial (when the maximum response rate was shared by multiple time bins, we assigned the peak to the median bin) and then computed the across trial mean peak location for each option and bird. Across birds, the mean peak locations occurred at 5.3 (SE: .59), 9.3 (SE: .89), 7.6 (SE: .83) and 16.5 s (SE: 1.38) for options A_5, B_{10}, C_{10}, and D_{20}, respectively. A paired-samples *t*-test revealed no significant differences in peak locations for options B_{10} and C_{10} [$t(6)=1.996$, $p=.093$]. These findings together with those from pecking rates confirm that birds were responding to the absolute properties of the options rather than to perceptually distorted properties.
