# Supplementary material for: Context-Dependent Preferences in Starlings: Linking Ecology, Foraging and Choice
Source: PLoS One. 2013 May 21;8(5):e64934. doi: 10.1371/journal.pone.0064934 (PMC3660320; doi:10.1371/journal.pone.0064934)
Supplement: Text S4 — Analyses of complete latency distributions. (PDF) [file pone.0064934.s006.pdf]

Text S4

Analyses of complete latency distributions. We summarized the latency distributions as cumulative frequency scores given by the area under the cumulative distribution plot (average cumulative distributions for each option are presented in Figure S1). Higher scores capture shorter latencies to respond because in such cases more latencies occur in earlier bins (e.g. the maximum score of 20 occurs when all latencies fall in the first of 20 bins). The interesting comparisons are between A_5 and C_{10} and between B_{10} and C_{10} as these options were involved in the preference tests. As suggested by Figure S1, the cumulative scores for option C_{10} were significantly larger than those found for option B_{10} [$t(6)=-2.699$, $p=.036$], coinciding with the analysis based on central tendency of the latencies. Also coinciding with the analysis of central trends, we found no significant differences in cumulative scores between A_5 and C_{10} [$t(6)=1.502$, $p=.184$], which goes against the predictions of the SCM and suggests that A_5's higher immediacy was counteracted by the options' common ranking or masked by a floor effect as described and discussed in the results and discussion sections, respectively.
